# 3D and 2D Metal Halide Perovskites for Blue Light-Emitting Diodes

**DOI:** 10.3390/ma15134571

**Published:** 2022-06-29

**Authors:** Min-Ho Park

**Affiliations:** Department of Organic Materials and Fiber Engineering, Integrative Institute of Basic Science, Soongsil University, 369 Sangdo-Ro, Dongjak-Gu, Seoul 06978, Korea; minhopark@ssu.ac.kr

**Keywords:** blue perovskite light-emitting diodes, metal halide perovskites, mixed-halide perovskites, quasi-2D perovskites, nanoplatelets

## Abstract

Metal halide perovskites (MHPs) are emerging next-generation light emitters that have attracted attention in academia and industry owing to their low material cost, simple synthesis, and wide color gamut. Efficient strategies for MHP modification are being actively studied to attain high performance demonstrated by commercial light-emitting diodes (LEDs) based on organic emitters. Active studies have overcome the limitations of the external quantum efficiencies (EQEs) of green and red MHP LEDs (PeLEDs); therefore, the EQEs of PeLEDs (red: 21.3% at 649 nm; green: 23.4% at 530 nm) have nearly reached the theoretical limit for the light outcoupling of single-structured planar LEDs. However, the EQEs of blue PeLEDs (12.1% at 488 nm and 1.12% at 445 nm) are still lower than approximately half of those of green and red PeLEDs. To commercialize PeLEDs for future full-color displays, the EQEs of blue MHP emitters should be improved by approximately 2 times for sky-blue and more than 20 times for deep-blue MHP emitters to attain values comparable to the EQEs of red and green PeLEDs. Therefore, based on the reported effective approaches for the preparation of blue PeLEDs, a synergistic strategy for boosting the EQE of blue PeLEDs can be devised for commercialization in future full-color displays. This review covers efficient strategies for improving blue PeLEDs using fundamental approaches of material engineering, including compositional or dimensional engineering, thereby providing inspiration for researchers.

## 1. Introduction

Metal halide perovskites (MHPs) have been extensively studied for various optoelectronic applications, such as solar cells [1,2], photodetectors [3], photocatalysts [4], lasers [5], and light-emitting diodes (LEDs) [6,7]. In particular, many research groups worldwide have reported various possibilities for the implementation of these light emitters, and as a result, MHPs have been established as next-generation LEDs [8,9,10,11,12,13,14]. MHP-based LEDs (PeLEDs) have easy bandgap tunability, low material cost, easy solution processability, high carrier mobility, and high color purity. In addition to these remarkable advantages, a significant efficiency improvement, comparable to conventional organic LEDs, has been demonstrated in a short time, which supports the claim that PeLEDs deserve the title of next-generation light emitters for display and solid-state lighting. However, compared with the device efficiencies of red and green PeLEDs (red: 21.3% at 649 nm; green: 23.4% at 530 nm), that of blue PeLEDs is quite low (12.1% at 488 nm and 1.12% at 445 nm) [15,16,17,18].

Blue is one of the primary colors of light, in addition to red and green. As demonstrated by the history of commercial inorganic LEDs, full-color LED displays consisting of the primary colors of light were developed after GaN-based blue LEDs. Moreover, the development of blue emitters has always been considered the most challenging research task in the development of novel displays because of the intrinsic limitations of blue emitters: high photon energy [19,20], low operational stability [21,22], and material instability [23,24]. Therefore, although PeLEDs have been actively studied for approximately 7 years, early interest in the development of blue PeLEDs may advance the development of promising perovskite displays. To achieve this goal, many research groups have attempted to develop strategies for efficient blue PeLED fabrication through direct compositional engineering [25,26,27], dimensional engineering [28,29], additive engineering of MHPs [30,31], and indirect modifications of adjacent functional layers, including hole- and electron-transporting layers [32,33,34].

This review focuses on the main material engineering strategies for the preparation of efficient blue PeLEDs based on mixed-halide and low-dimensional MHPs. Moreover, this review presents promising strategies used for the development of 3D and 2D blue PeLEDs and covers the scientific background behind them to help provide a systematic understanding. In the first part, the electronic structures of mixed-halide perovskites and cation engineering for efficient mixed-halide blue PeLEDs are reviewed. The second part covers efficient strategies for low-dimensional perovskites based on ligand engineering, phase engineering, and surface defect engineering. In the final section, we briefly summarize promising strategies for 3D and 2D blue PeLED manufacturing.

## 2. Mixed-Halide Perovskites

### 2.1. Electronic Structures

**Figure 1:**
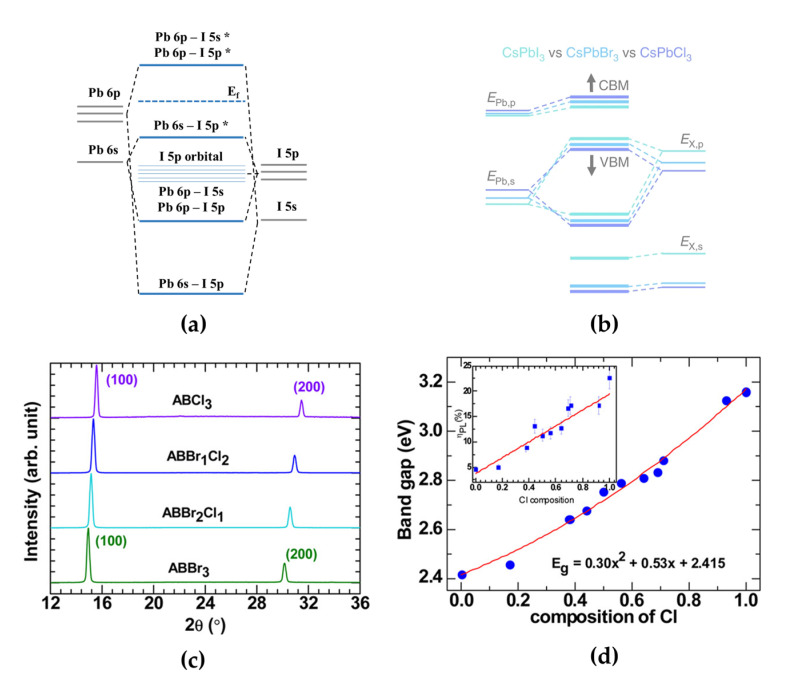
(**a**) Electronic band structures of [PbI_6_]^−4^ (* antibonding orbitals). (**b**) Schematic energy levels in CsPbX_3_ perovskites with different halide anions, calculated using tight-binding analysis. Reprinted with permission from [27], Copyright 2019 Springer Nature. (**c**) X-ray diffraction (XRD) patterns of AB(Br_1−x_Cl_x_)_3_ films (x = 0, 0.33, 0.66, and 1). Reprinted with permission from [39], Copyright 2016 American Chemical Society. (**d**) Quadratic behavior of AB(Br_1−x_Cl_x_)_3_ bandgaps as a function of Cl content. Reprinted with permission from [37], Copyright 2015 American Chemical Society.

A simple approach to adjusting the electroluminescence (EL) spectrum of PeLEDs is the compositional engineering of the X-site halide ions in APbX_3_ MHPs. The electronic band structures of single-halide-based 3D MHPs are determined by the orbital states of their elements (Figure 1a) [35]. The highest valence band consists of Pb 6*s* orbitals and single-halide 5*p* orbitals (mainly attributed to halide *p* orbitals), and the lowest conduction band consists of Pb 6*p* orbitals and single-halide 5*p* orbitals (mainly attributed to Pb *p* orbitals) because of partial hybridization and spin–orbital coupling (Figure 1b) [9,27,36]. Therefore, APbCl_3_, APbBr_3_, and APbI_3_ (A = CH_3_NH_3_ (methylammonium, MA), CH(NH_2_)_2_ (formamidinium, FA), Cs) have shown various light emission colors depending on the valence band maximum (VBM) and conduction band minimum (CBM) formations [8,10]. However, pure blue-emitting MHPs with an emission wavelength of approximately 465 nm are intrinsically difficult to synthesize using only single-halide 3D MHPs. Pure chloride-containing 3D MHPs exhibit a deep blue-violet emission spectrum (~409 nm) with a wide bandgap (3.16 eV) [37,38].

Mixing halide anions with MHPs can effectively change their structural, optical, and electronic properties. When other halide ions are doped into single-halide MHPs, the difference in the ionic radii of halide ions (Cl^−^ < Br^−^ < I^−^) induces changes in the lattice constant with lattice distortion, which leads to a change in the distance between Pb and halide atoms [36]. Therefore, the electronic interactions between Pb and halide ions can also be affected, and thus, band structures can be effectively tuned because the PbX_6_ octahedral cage is important for determining the electronic properties of MHPs [39]. It has been theoretically proven that a Cl atom, which is smaller than a Br atom, can induce a larger distance change with Pb atoms when it is doped into iodine MHPs [36]. In Br- or Cl-doped MAPbI_3_, each VBM is affected by the mixed contributions of I 5*p* orbitals, with each Br 4*p* orbital or Cl 3*p* orbital, for identical halide doping ratios of 0.67, whereas CBMs are not affected by doped halogen atoms.

For pure blue light-emitting MHPs, the electronic bandgaps of MHPs can be tuned when smaller Cl atoms are doped into bromine MHPs or larger Br atoms are doped into chlorine MHPs to synthesize Br–Cl mixed-halide perovskites, which are promising pure blue light emitters. Kumawat et al. experimentally and theoretically investigated the structural, optical, and electronic property changes in MAPb(Br_1−x_Cl_x_)_3_ [39]. With an increase in the Cl ratio in MAPb(Br_1−x_Cl_x_)_3_, the X-ray diffraction (XRD) peaks of MAPb(Br_1−x_Cl_x_)_3_ gradually shifted towards higher diffraction angles (Figure 1c). This XRD peak shift indicates that smaller Cl atoms deform the crystal lattice, and thus, the experimental lattice parameter linearly decreases with increasing Cl content (MAPb(Br_1−x_Cl_x_)_3_ lattice parameters: 5.95 Å [x = 0], 5.83 Å [x = 0.33], 5.75 Å [x = 0.66], and 5.66 Å [x = 1]). Moreover, a full width at half maximum (FWHM) of the XRD peaks in mixed-halide perovskites was slightly broader than in single-halide perovskites owing to crystallinity changes. They also proved the origin of bandgap tuning in mixed-halide perovskites by calculating the formation of VBM and CBM. After adding Cl at a 0.66 ratio to Br–Cl mixed-halide perovskites, the Cl 3*p* orbital state led to a mixed contribution of Br 4*p* and Cl 3*p* states at the VBM, while the contribution of the Pb 6*p* orbital state at the CBM was fixed. The emission spectrum depending on the ratio between the X-site Br and Cl halide ions can be easily controlled from the green (0% Cl composition) to the blue (100% Cl composition) wavelength region. Therefore, precise stoichiometric control of Br and Cl atoms in mixed-halide perovskites is an effective method that can intrinsically achieve blue emission spectrum through electronic modification.

Typically, PeLEDs have adopted a planar-type multilayered device structure consisting of electrodes, MHP EMLs, and other functional common layers for efficient charge injecting and transporting properties. The electrons and holes injected from the cathode and anode under the electric field are transported through the hole transport layer (HTL) and electron transport layer and then confined in MHP EMLs to form excitons before light emission. Moreover, device characteristics, such as current efficiency, external quantum efficiency (EQE), and brightness, are highly dependent on the radiative recombination rate originating from the quality of the MHP thin films because the MHPs have defects, such as halide vacancies and grain boundaries, which act as charge trapping sites during device operation [40,41]. Kumawat et al. reported the first blue PeLED using Br–Cl mixed-halide perovskite as emitting layers (EMLs) [37]. To synthesize Br–Cl mixed-halide perovskite solutions and fabricate blue PeLEDs, they prepared MAPbBr_3_ and MAPbCl_3_ solutions and mixed them at different volumetric ratios. MAPb(Br_1−x_Cl_x_)_3_ solutions were then spin-coated onto poly(3,4-ethylenedioxythiophene):poly(styrenesulfonate) (PEDOT:PSS)/ITO glass substrates in a nitrogen-filled glove box to form EMLs. The deposited MAPb(Br_1−x_Cl_x_)_3_ [0 ≤ x ≤ 1] films have a cubic crystal structure at room temperature. Cl content in mixed-halide perovskites can broaden the bandgap from 2.4 eV for green light-emitting MAPbBr_3_ to 2.5–2.75 eV for blue light-emitting MAPb(Br_1−x_Cl_x_)_3_ (Figure 1d). Moreover, the PL efficiency increased from ~5%–25% because the phonon-assisted nonradiative decay channel was less dominant. They fabricated the first blue PeLED using compositionally controlled MAPbBr_1.08_Cl_1.92_. The calculated bandgap, exciton peak, and PL peak positions of MAPbBr_1.08_Cl_1.92_ (Cl composition = 0.64) are 2.81, 2.75, and 2.63 eV, respectively. In contrast to the exciton peak, the red-shifted PL peak was derived from structural disorder, and the EL peak was further red-shifted to 482 nm (~2.57 eV) because the injected charge carriers were trapped in lower-energy states. Furthermore, the wide bandgap of MAPbBr_1.08_Cl_1.92_ yielded poor electrical properties in blue PeLEDs. These poor electrical properties originating from the MAPbBr_1.08_Cl_1.92_ EML resulted in poor device performance, mainly owing to a high hole-injection barrier, although the PL efficiency was higher than that of the green-emitting MAPbBr_1.86_Cl_1.14_ film.

### 2.2. Cation Engineering

**Figure 2:**
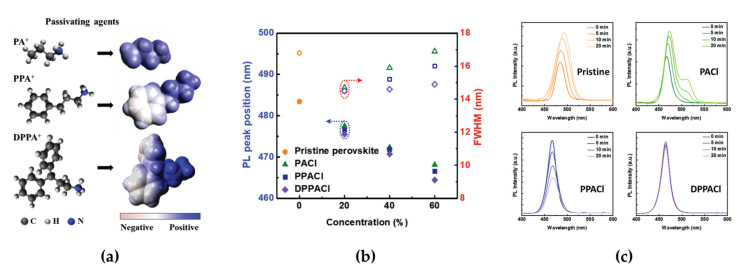
(**a**) Molecular structures and electrostatic potentials of PA^+^, PPA^+^, and DPPA^+^. (**b**) Photoluminescence (PL) peak positions (left axis, filled symbols) and full width at half maximum (right axis, unfilled symbols) of perovskites as a function of the passivating agent concentration for the different passivating agents. (**c**) Steady-state PL spectra of pristine and 60% PACl, 60% PPACl, and 60% DPPACl perovskite films. Reprinted with permission from [45], Copyright 2020 John Wiley and Sons.

Mixed-halide perovskites can be obtained via simpler synthesis routes, such as simple precursor mixing or anion exchange methods, compared with other types of blue-emissive perovskites reviewed in this paper [37,39,42]. However, one intrinsic limitation of mixed-halide perovskites is phase segregation through halide ion migration during device operation or light illumination [43,44,45,46]. The intrinsic operational instability originating mainly from halide ion migration can limit practical LED application in next-generation displays; therefore, overcoming stability issues of mixed-halide perovskites applied in blue emitters is an important challenge for researchers to resolve. Kim et al. reported a defect passivation method by developing organic ammonium cations as passivating agents, such as propylammonium (PA^+^), phenylpropylammonium (PPA^+^), and diphenylpropylammonium (DPPA^+^), to overcome the phase instability in blue PeLEDs (Figure 2a) [45]. The organic ammonium cations in passivating agents have different passivation effects on Cl^−^ vacancies in mixed-halide perovskites depending on the number of benzene rings (PA^+^: 0, PPA^+^: 1, DPPA^+^: 2). DPPA^+^ containing two benzene rings effectively generates an unbalanced electron distribution, which can induce polarized organic ammonium cations. In this regard, highly polarized DPPA^+^ can have strong electrostatic interactions with halide anions (Cl anion in this report), which can adsorb onto the perovskite surface defects. As a result, DPPA^+^ shows effective chemical interactions with Cl^−^ vacancies in CsPbBr_2_Cl, thus suppressing halide migration in CsPbBr_2_Cl. In addition to the effect of the number of benzene rings in the ammonium cations, the concentration of the passivating agents significantly affects the passivation capability of CsPbBr_2_Cl. The PL spectrum of CsPbBr_2_Cl with passivating agents showed a trend that depended on both the number of benzene rings and the concentration of the passivating agents (Figure 2b). With increasing numbers of benzene rings, the PL peak was blue-shifted from 468 to 464 nm, and the FWHM of the PL spectrum decreased from 16.9 to 14.9 nm. Moreover, blue-shifted PL peaks were observed for all passivating agents, with a high concentration of 60% because of the efficient adsorption of Cl anions on the perovskite surface. The DPPACl passivating agent also affected the operational stability in terms of halide migration. The steady-state PL spectrum change, such as a shift in the peak position and a decrease in intensity over time in DPPACl, rarely appeared when compared with PACl or PPACl (Figure 2c). The measured PL quantum yield (PLQY) further supported the passivation effect (11.6% for PACl, 15.7% for PPACl, and 20.4% for DPPACl). These PL properties clearly show the strong passivation effect of 60% DPPACl on spectral stability through the suppression of halide migration. The better passivation effect of DPPACl, which is proven by the PL characteristics, was similarly observed in the EL characteristics of PeLEDs. The maximum luminance and EQE of optimized PeLEDs for 60% DPPACl were improved to 442 cd/m^2^ and 1.92%, respectively (90.8 cd/m^2^ and 0.22% for 60% PACl and 321.1 cd/m^2^ and 0.86% for 60% PPACl). In terms of the operational stability of PeLEDs, the EL peak position (centered at 464 nm) with the increase in applied bias remained below 8 V and then slightly red-shifted above 9 V because of ion migration owing to the effects of high electric field, moisture, oxygen, and Joule heating. As a result, the device half-lifetime was improved by more than four times (~420 s).

**Figure 3:**
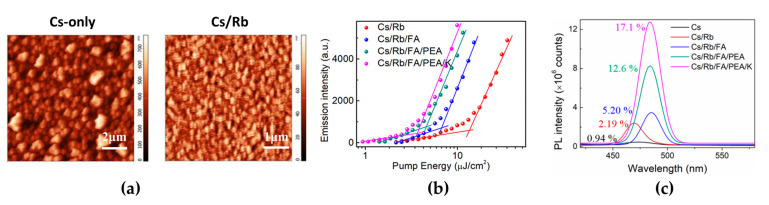
(**a**) Atomic force microscopy images of the CsPb(Cl/Br)_3_ (RMS = 113 nm) and (Cs/Rb)Pb(Cl/Br)_3_ (RMS = 13.3 nm) films. (**b**) Integrated PL emission intensity of different perovskite films as a function of laser pumping energy. (**c**) PL spectra and PL quantum yields (PLQYs) of APb(Cl/Br)_3_ (A = multiple cations of Cs, Rb, formamidinium (FA), phenylethylammonium (PEA), and K) films. Reprinted with permission from [48], Copyright 2020 American Chemical Society.

Mixed-halide perovskites usually exhibit inhomogeneous film formation with poor surface morphology owing to their fast crystallization [37,47]. The poor film quality causes poor PL properties and acts as a leakage current path in PeLEDs. A-site cation engineering is effective for improving the morphological, structural, and optical properties of mixed-halide perovskites. A-site cations, such as Rb, FA, phenylethylammonium (PEA), and K doped into mixed-halide perovskites, exert different effects [48]. In contrast to other cations, Rb cations showed a unique effect on the surface coverage, which presented continuous small grains, whereas the undoped CsPb(Cl/Br)_3_ film formed rectangular cube-shaped and sparsely grown large crystals with an extremely rough surface morphology (surface coverage: 62% for Cs-only, 88% for Cs/Rb; root mean square: 113 nm for Cs-only, 13.3 nm for Cs/Rb) (Figure 3a). The measured XRD peaks clearly show that the Rb-doped Cs film has stronger (100) and (200) peaks than the Cs-only and other cation-doped Cs films. K cations, which are smaller than Cs cations, can occupy the interstitial sites rather than substitute Cs, but larger FA cations expand the crystal lattice by entering the crystals. At the same time, large PEA cations are only located at the crystal surface; thus, the pristine crystal structure of CsPb(Cl/Br)_3_ did not change, although 2D perovskites could be formed. Moreover, grazing-incidence wide-angle X-ray scattering (GIWAXS) measurements demonstrated the Rb cation doping effect on the crystallization behavior: preference for orientational crystal growth. Moreover, Rb cations improved the PL properties, which showed the passivation effect (PLQY and PL lifetime: 0.94% and 3.61 ns for Cs-only, respectively, and 2.19% and 5.09 ns for Cs/Rb). Therefore, Rb cations play a significant role in modifying the crystallization kinetics and improving the surface morphology and PL properties. In addition to Rb cation doping, multiple-cation (FA, PEA, K) doping can be introduced to further improve the film morphology and optical properties [47,48,49]. Doping other cations (FA, PEA, K) at low concentrations can effectively reduce pinholes, grow small grains without noticeable changes to crystallinity, and passivate surface defects. FA cation doping induced a red shift in the PL peak, with shifts in the valence band (VB) and conduction band (CB) levels (PL peak positions: 473 nm for Cs-only, 470 nm for Cs/Rb, 484 nm for Cs/Rb/FA). PEA and K cations are mainly located at the crystal surfaces and passivated surface vacancy defects; thus, PL emission was observed at a low excitation energy (Figure 3b). Therefore, the improved surface morphology and passivation effect caused by multiple cation doping significantly enhanced the PLQY (by up to 17.1%). The additional effect of cation engineering is the ability to tune the energy levels, which can improve charge balance in PeLEDs. By elevating the levels of VB and CB, the hole injection barrier from anode to EMLs was reduced, and thus, the maximum luminance and EQE of fabricated PeLEDs were improved from 13.8 cd/m^2^ and 0.0037% to 4015 cd/m^2^ and 2.01%, respectively. In particular, additional doping of Rb-doped CsPb(Cl/Br)_3_ with FA, PEA, and K cations dramatically improved the operational device half-lifetime (20 min for Cs/Rb, 300 min for Cs/Rb/FA/PEA/K). To synthetically improve the characteristics of mixed-halide perovskite films and devices, multiple-cation engineering can provide rational design strategies.

**Figure 4:**
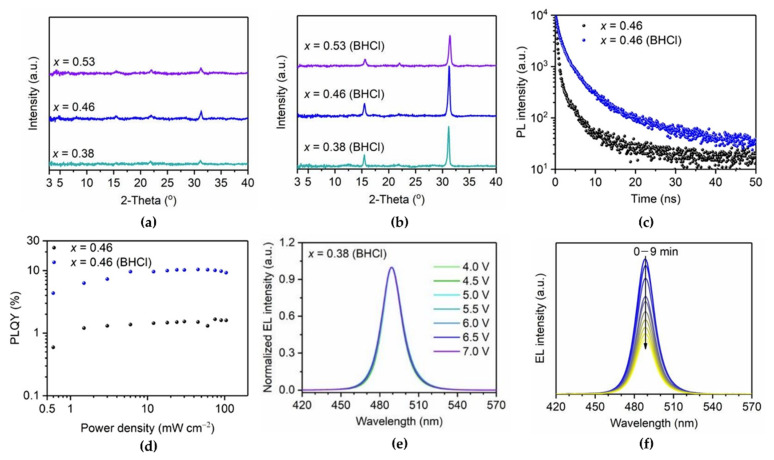
XRD patterns of (**a**) CsPb(Br_1−x_Cl_x_)_3_ and (**b**) benzamidine hydrochloride (BHCl)-treated CsPb(Br_1−x_Cl_x_)_3_ films with varying *x*. (**c**) PL decay curves and (**d**) power-dependent PLQYs of control and BHCl-treated CsPb(Br_0.54_Cl_0.46_)_3_ films. (**e**) Normalized electroluminescence (EL) spectra of PeLEDs with *x* = 0.38 (BHCl) film. (**f**) EL spectra of PeLED with *x* = 0.38 (BHCl) under a constant current density of 5 mA/cm^2^. Reprinted with permission from [44], Copyright 2021 American Chemical Society.

Organic ammonium chloride salts are also good candidates for effectively passivating surface defects at grain boundaries to enhance the spectral stability of 3D blue PeLEDs [44]. Yu et al. developed benzamidine hydrochloride (BHCl) as an additive for defect passivation, without changing the crystal structure or forming other phases. BHCl effectively passivated defects through the interaction between the C=N group in BHCl and uncoordinated Pb atoms on the crystal surface. The XRD results clearly show that BHCl significantly increased the main peak intensities at the (100) and (200) planes in all BHCl-treated samples (Figure 4a,b). GIWAXS showed highly oriented crystals of BHCl-treated CsPb(Br_1−x_Cl_x_)_3_ with high crystallinity. In terms of the optical properties, after the introduction of BHCl into CsPb(Br_1−x_Cl_x_)_3_, the PL peak was slightly red-shifted and became narrower owing to the defect passivation effect. Moreover, the PL lifetime of the BHCl-treated CsPb(Br_1−x_Cl_x_)_3_ film increased, and the PLQY also improved to 10.4% (Figure 4c,d). These enhanced optical properties can be simultaneously attributed to better crystallinity and reduced defect density. The reduced defect density at the grain boundary could suppress ion migration, and thus, the incorporated BHCl improved operational stability, including spectral stability and device lifetime. It was confirmed that the EL peak positions of the fabricated PeLEDs were maintained during the lifetime measurements (Figure 4e,f).

## 3. Low-Dimensional Perovskites (LDPs)

Another major strategy for blue-emitting MHPs for blue LEDs is the synthesis of low-dimensional single-halide MHPs, such as quasi-2D, nanoplatelet (NPL), and nanocrystal perovskites. LDPs rely on the quantum confinement effect of nanoscale crystals that are close to the exciton Bohr radius, thereby exhibiting a blue emissive spectrum [50,51,52,53]. LDPs have several advantages over 3D mixed-halide MHPs. Blue-emitting LDPs do not necessarily require the use of mixed-halide MHPs, which suffer from operational stability issues due to phase segregation. Moreover, LDPs with smaller crystals can exhibit larger exciton binding energies than organic emitters (~300 meV) and wider bandgaps than 3D bulk MHP crystals owing to the quantum and structural effects of lattice strain at the crystal surface [50,52]. The large organic ligands, including the aliphatic and phenyl groups, can segregate at the crystal surface to form a 2D crystal structure. LDPs can lead to strong exciton localization within crystals, which is promising for LED applications [53].

### 3.1. Ligand Engineering

**Figure 5:**
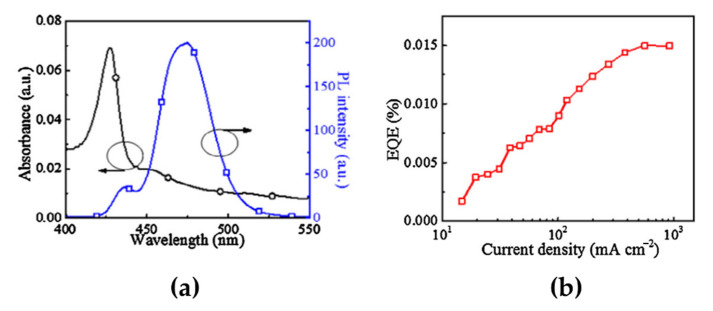
(**a**) Absorption (left axis) and PL spectra (right axis) of the quasi-2D PCPbB perovskite film consisting of 2D (4-PBA)_2_PbBr_4_ and 3D CsPbBr_3_. (**b**) External quantum efficiencies (EQEs) as a function of current density. Reprinted with permission from [58], Copyright 2016 Elsevier.

A simple method for LDP synthesis is to introduce large organic ligands, such as butylammonium (BA), PEA, and phenylbutylammonium bromide (PBA), into the MHP precursor solutions to form 2D-phase-containing MHP films [54,55,56]. By precisely controlling the concentration of large organic ligands, which is the molecular ratio between organic ligands and MHP precursors, the emission spectrum of MHPs can be tuned from the green to the blue wavelength region [57]. Another important factor for LDP synthesis is the chemical interaction between the organic ligands and crystal surfaces. Cheng et al. used 4-phenylbutylamine (4-PBA) as a ligand in CsBr and PbBr_2_ precursor solutions at a molar ratio of 2:1:1 [58]. Spin-coated 4-PBA-added CsPbBr_3_ films contained intermediate phases: quasi-2D MHPs consisting of 2D (4-PBA)_2_PbBr_4_ and 3D CsPbBr_3_. Exciton absorption peaks were observed at 427 and 455 nm, without any absorption peaks originating from the 3D phases (Figure 5a). Moreover, a strong PL peak was detected at 475 nm, which was blue-shifted by approximately 52 nm compared with the PL peak position in 3D CsPbBr_3_ (~527 nm) (Figure 5a). Therefore, measuring changes in the optical properties can be an easy and efficient method for defining the formation of quasi-2D MHPs. Furthermore, the fabricated quasi-2D blue PeLEDs showed a main EL peak at 491 nm, which did not change with increasing voltage. The maximum EQE was 0.015% at the maximum luminance of 186 cd/m^2^ (Figure 5b). Compared with mixed-halide perovskites that have a phase segregation problem reviewed in the previous chapter, LDP emitters have a higher spectral stability. However, the PL and EL spectra of the quasi-2D MHPs showed multiple emission peaks owing to the mixed phases. As mentioned above, the ratio between the 2D and 3D phases mainly depends on the concentration of large organic ligands. Therefore, careful compositional engineering should be considered for quasi-2D blue PeLEDs. Furthermore, when quasi-2D MHPs are synthesized using APbBr_3_ (A = Cs, MA) with large organic ligands, a green EL spectrum is predominantly observed because of the energy transfer from LDPs with a larger bandgap to 3D CsPbBr_3_ with a smaller bandgap [59,60]. For this reason, the color stability and purity of PeLEDs are limited by multiple emission peaks.

**Figure 6:**
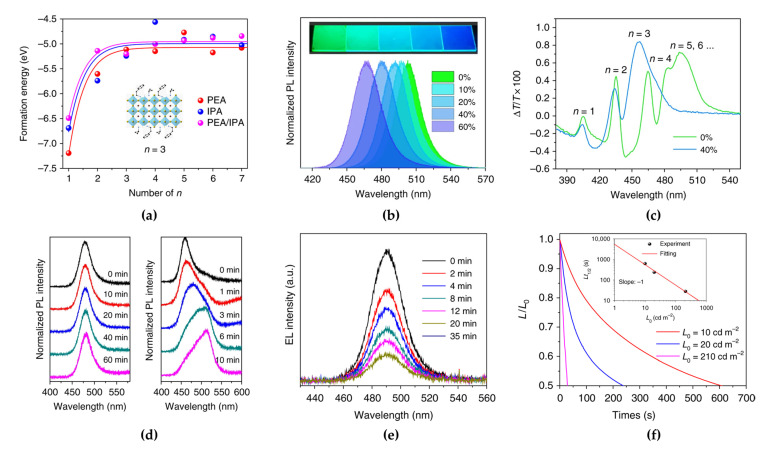
(**a**) Calculated formation energy of PEABr and isopropylammonium (IPA)Br-mixed quasi-2D perovskites with different *n* values (inset: PEA/IPA-mixed quasi-2D perovskite with *n* = 3). (**b**) PL spectra of PEA_2_A_1.5_Pb_2.5_Br_8.5_ with 0−60% IPABr. (**c**) Transient absorption (TA) spectra of PEA_2_A_1.5_Pb_2.5_Br_8.5_ with 0% and 40% IPABr. (**d**) PL spectra of quasi-2D perovskite (left) and MAPbCl_1.5_Br_1.5_ (right) films under continuous laser radiation (325 nm, 7 W/cm^2^) at different exposure times. (**e**) EL spectra of PeLED vs. operation times. (**f**) Device half-lifetime of PeLEDs vs. different initial luminance (inset: estimated half-lifetime by stretched exponential decay). Reprinted with permission from [61], Copyright 2018 Springer Nature.

Xing et al. reported the ligand combination method of a conventional PEA ligand with isopropylammonium (IPA) as a shorter ligand to improve the optical properties by controlling the phase formation energy [61]. The IPA ligand reduces the van der Waals interactions between large organic ligands, thus destabilizing the *n* = 1 phase and inducing slow crystallization. Density functional theory (DFT) simulations showed that depending on the type of ligand, the formation energy of the 2D perovskite (*n* = 1) phase can be changed owing to the partial destabilization of the *n* = 1 phase (Figure 6a). With increasing IPA concentration, the PL peaks significantly blue-shifted from 504 nm with 0% IPA to 467 nm with 60% IPA in quasi-2D PEA_2_(MA_1−x_Cs_x_)_1.5_Pb_2.5_Br_8.5_ perovskites (Figure 6b). The effect of IPA addition on the optical properties was further investigated by analyzing the photogenerated carrier dynamics using TA. The observed bleaching peaks in the TA results of quasi-2D perovskites are related to the presence of multiple phases. In contrast to the pristine PEA_2_(MA_1−x_Cs_x_)_1.5_Pb_2.5_Br_8.5_ film, the 40% IPA-added film showed four distinct TA peaks corresponding to low-dimensional phases (*n* ≤ 4), without the TA peak corresponding to bulk phases (*n* ≥ 5) (Figure 6c). Moreover, the PL spectrum of the 40% IPA-added PEA_2_(MA_1−x_Cs_x_)_1.5_Pb_2.5_Br_8.5_ film did not change under 325 nm laser irradiation at different exposure times, while that of the bulk mixed-halide perovskite (MAPbBr_1.5_Cl_1.5_) significantly red-shifted (Figure 6d). The large organic ligands positioned at the crystal surface have insulating properties that reduce the current density of LEDs. Unlike in 3D bulk PeLEDs, in LDP-based PeLEDs, the thickness of EMLs should be considered to optimize the electron–hole balance and surface morphology of the EMLs. Therefore, the fabricated blue PeLEDs using IPA-added quasi-2D perovskites with an optimized 80 nm-thick EML showed an EL peak at 490 nm, and maximum device efficiencies of 1.5%, 0.92 lm/W, and 2.8 cd/A were achieved. Moreover, the enhanced spectral stabilities, such as peak positions and FWHM of the EL spectrum depending on the applied voltages, were maintained without shifts or changes, as observed in PL stability, depending on the light exposure. However, the EL intensity continuously decreased over time under a constant voltage (Figure 6e). This low operational stability is strongly related to the short device lifetime, which ranged from a few seconds to minutes (Figure 6f).

**Figure 7:**
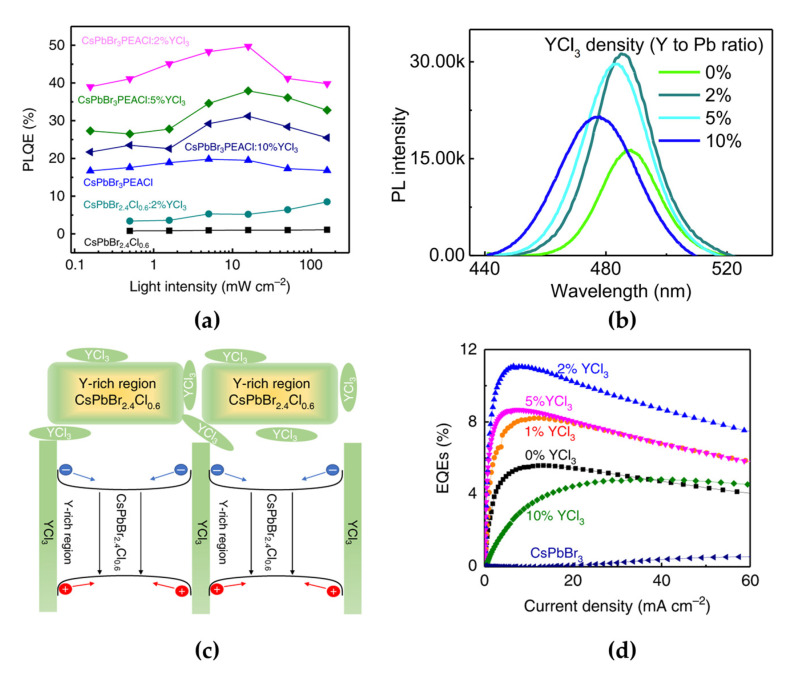
(**a**) Laser-power-dependent PL quantum efficiencies (PLQEs) of perovskites with different compositions. (**b**) PL spectra of CsPbBr_3_:PEACl (1:1) films with different YCl_3_ ratios. The PL peak positions are at 487 nm (0% YCl_3_), 485 nm (2% YCl_3_), 483 nm (5% YCl_3_), and 477 nm (20% YCl_3_). (**c**) Schematic of Y distribution in the CsPbBr_3_:PEACl (1:1) film and its bandgap around the grain surface. (**d**) EQEs as a function of current density of CsPbBr_3_-based PeLEDs. Reprinted with permission from [62], Copyright 2019 Springer Nature.

To overcome the limited long-term operational stability, Wang et al. introduced YCl_3_ in both single-halide quasi-2D MHPs (PEACl:CsPbBr_3_ with a 1:1 molar ratio) and mixed-halide 3D MHPs (CsPbBr_2.4_Cl_0.6_) to improve the stability of blue PeLEDs [62]. The measured PL peak positions and maximum PL quantum efficiencies (PLQEs) were 487 nm and 1.1% for 3D CsPbBr_2.4_Cl_0.6_ and 486 nm and 19.8% for quasi-2D PEACl:CsPbBr_3_, respectively (Figure 7a). The higher PLQE of PEACl:CsPbBr_3_ originated from the strong quantum confinement effect in the 2D phases. As reviewed above, the emission spectrum of quasi-2D PEACl:CsPbBr_3_ can be tuned from the green to the blue wavelength region by controlling the ratio between PEACl and CsPbBr_3_. In addition, the YCl_3_ introduction also affected the spectral shift in the short-wavelength region (Figure 7b). Furthermore, the concentration of YCl_3_ was also critical to the PL efficiency of the quasi-2D perovskite film, owing to the effect of YCl_3_ cluster formation. Moreover, YCl_3_ effectively improved surface coverage and morphology, which effectively reduced the leakage current of the PeLEDs. Incorporated Y gets accumulated on the film surface and increases the bandgap of CsPbBr_3_, thereby forming a crystal surface with a wider bandgap through Pb ion replacement and Cs ion vacancy formation. As a result, Y^3+^-incorporated CsPbBr_3_ grains can effectively confine charge carriers inside the grains (Figure 7c) and reduce the density of nonradiative recombination centers that originate from defective grain boundaries. Consequently, the PLQE increased to 49.7% with an optimized doping concentration of 2% YCl_3_ in PEACl:CsPbBr_3_ (1:1) (Figure 7a). The blue PeLEDs fabricated using 2% YCl_3_-doped PEACl:CsPbBr_3_ showed the highest EQE with a value of 11% and a maximum luminance of 9040 cd/m^2^ (Figure 7d). In particular, the effect of YCl_3_ doping on quasi-2D PeLEDs was clearly demonstrated over the device lifetime.

### 3.2. Phase Engineering

For the efficient route of quasi-2D MHP synthesis, it was confirmed that large organic ligands significantly affect the relative phase formation mechanism of the 3D and 2D phases and the luminescence efficiencies of PLQEs and EQEs. Although large organic ligands control the formation of bulk and low-dimensional phases, precise control of multiple phases inside the quasi-2D MHP thin films can further improve device efficiency by suppressing the severe nonradiative recombination caused by strong exciton–phonon coupling in the 2D *n* = 1 phase and cascade energy transfer to the smaller bandgap [53,59,63,64,65,66]. In particular, the formation energy of the *n* = 1 phase is lower than that of *n* ≥ 2 phases; therefore, controlling the *n* = 1 phase formation can be an efficient strategy.

**Figure 8:**
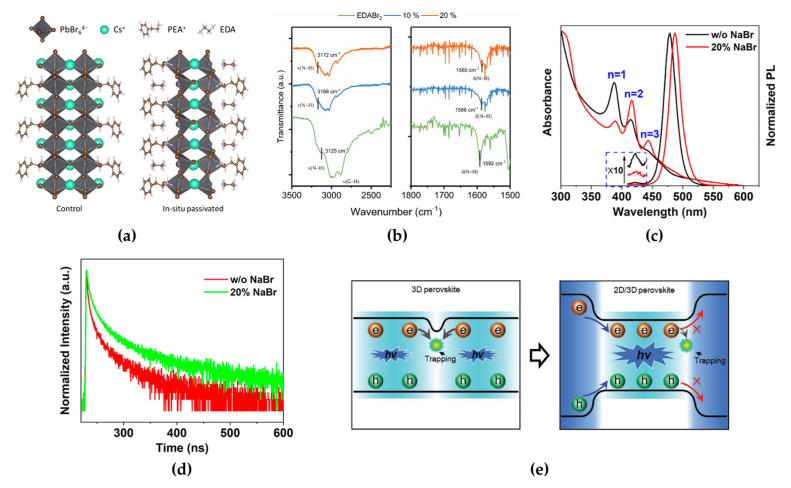
(**a**) Structures of controlled and passivated quasi-2D (*n* = 2) perovskites. Reprinted with permission from [63], Copyright 2020 American Chemical Society. (**b**) Fourier-transform infrared (FTIR) absorption spectra of thin films (*ν*(N−H), *ν*(C−H): N−H and C−H bond stretching; *δ*(N−H): N−H scissoring). Reprinted with permission from [63], Copyright 2020 American Chemical Society. (**c**) Absorption and PL spectra of quasi-2D perovskites with rearranged phase distribution. Reprinted with permission from [64], Copyright 2020 American Chemical Society. (**d**) PL decay with a 375 nm excitation wavelength for quasi-2D perovskite films with and without 20% NaBr. Reprinted with permission from [64], Copyright 2020 American Chemical Society. (**e**) Schematic of carrier recombination dynamics depending on 3D (**left**) and 2D/3D (**right**) structures. Reprinted with permission from [65], Copyright 2020 John Wiley and Sons.

Worku et al. reported phase control methods by adopting diammonium salts that modify the surface energy of quasi-2D phases and by suppressing the formation of small-bandgap quasi-2D and 3D phases [63]. Blue-shifted UV–VIS absorption and PL spectra (from 490 to 454 nm) of ethylenediammonium dibromide (EDABr_2_)-substituted PEABr:CsPbBr_3_, which resulted from energy funneling, indicated an absence of small-bandgap phases, which have a green wavelength spectrum. This effect can be ascribed to the interaction between the nitrogen atoms in EDABr_2_ and undercoordinated Pb^2+^ ions at the crystal surface (Figure 8a). Moreover, the defect passivation effect of EDABr_2_ at the crystal surface was observed through the increased PL decay time in time-resolved PL (from 40 to 92 ns) and PLQE (from 55% to 75%), and the origin of the interaction of EDABr_2_, N-H stretching band shift, was observed using FTIR measurements (Figure 8b). The incorporation of small alkali metal cations, such as Li^+^ and Na^+^, which can act as inorganic spacers, is also an effective method for managing the phase distribution in quasi-2D MHP films [31,67,68]. Small alkali metal cations and large organic ligands can occupy the A-sites on the MHP surfaces. The incorporated alkali metal cations can decrease the thermodynamic stability of the *n* = 1 phase, thereby controlling the formation energy of the low-dimensional phases. When 20% sodium bromide (NaBr) was added to PEABr:CsPbBr_3_, the intensities of the absorption and PL peaks corresponding to the *n* = 1 phase decreased, whereas those of the *n* = 2 and *n* = 3 phases increased (Figure 8c) [64]. These changes were ascribed to the rearrangement of low-dimensional phases through the change in crystallization kinetics. The calculated formation energies of the 2D perovskites were −0.109 eV for PEA_2_PbBr_3_Cl, −0.008 eV for PEA_1.75_Na_0.25_PbBr_3_Cl, and 0.821 eV for Na_2_PbBr_3_Cl, thus theoretically supporting unstable crystal formation when Na cations were added to 2D perovskites. A suppressed *n* = 1 phase ratio in the rearranged low-dimensional phases can effectively reduce nonradiative recombination despite having the highest exciton binding energy; therefore, the incorporated 20% NaBr allowed for a relatively larger amount of excitons in the *n* = 2 phase so that energy could be transferred to larger-*n* phases. This effect improves the radiative recombination rate, as described in the time-resolved PL decay results (Figure 8d). The average PL lifetime of 30% NaBr-added PEABr:CsPbBr_3_ was increased to 45.1 ns (22.8 ns for PEABr:CsPbBr_3_ without NaBr). The modified phase distribution in quasi-2D MHP films has shown efficient energy cascade characteristics from the *n* = 2 phase to the 3D phase because of bandgap differences [65]. The effectively rearranged phase distribution not only facilitates efficient energy transfer from the 2D to 3D phases but also transports charge carriers through the reconstructed electronic band structures. Moreover, these rearranged phases facilitate charge carrier confinement in the smaller bandgap of the 3D phase, which can increase the probability of exciton formation by increasing the charge carrier density, thereby enhancing radiative recombination and device performance (Figure 8e).

**Figure 9:**
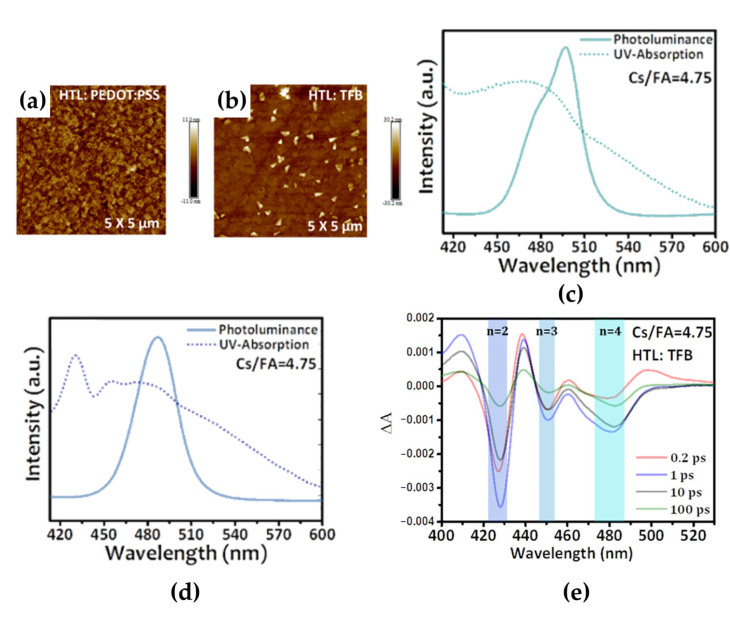
Atomic force topology of Cs/FA = 4.75 perovskite films on (**a**) poly(3,4-ethylenedioxythiophene):poly(styrenesulfonate) (PEDOT:PSS) and (**b**) poly[(9,9-dioctylfluorenyl-2,7-diyl)-co-(4,4-(N-(4-sec-butylphenyl)diphenylamine)] (TFB) substrates. Ultraviolet–visible absorption and PL spectra of perovskite films with different precursor stoichiometry on (**c**) PEDOT:PSS and (**d**) poly(9-vinylcarbazole) (PVK) substrates. (**e**) TA spectra using different probing timescales. Reprinted with permission from [69], Copyright 2021 American Chemical Society.

Another approach to controlling the distribution of the 2D and 3D phases in the films is proper selection of the underlying layer. PEDOT:PSS, poly[(9,9-dioctylfluorenyl-2,7-diyl)-co-(4,4-(N-(4-sec-butylphenyl)diphenylamine)] (TFB), poly(9-vinylcarbazole) (PVK), and poly[bis(4-phenyl)(4-butylphenyl)amine] (poly-TPD) have been used as HTLs in LED device architectures [69,70,71,72]. Moreover, most of the MHP layers were directly deposited on the PEDOT:PSS layer through solution processing. Therefore, the crystallization kinetics of MHP crystal growth during solution processing was significantly affected by the underlying layer [69,70]. Cai et al. reported that when TFB was used as the HTL, lower-dimensional 2D phases (2 ≤ *n* ≤ 4) were formed more favorably than when PEDOT:PSS was used as the HTL [69]. The microstructure images of the quasi-2D MHP films deposited on the PEDOT:PSS and TFB layers showed different morphological characteristics (Figure 9a,b). The smooth and low-roughness morphology with small grains was formed in the PBA_2_(APbX_3_)_1.74_PbX_4_ (A: Cs/FA = 4.75, X: Br/Cl = 6.6) film deposited on the PEDOT:PSS layer, whereas a relatively rough and island-like surface morphology with large grains was formed in the case of the film deposited on the TFB layer because of the higher density of the 3D phase on the surface. The different phase distributions exhibited by the films, depending on the underlying layers, were observed by measuring their optical properties. The quasi-2D perovskite film on the PEDOT:PSS layer showed a relatively broad ultraviolet−visible absorption spectrum, and a PL emission peak appeared at 496 nm (Figure 9c). These optical properties usually can be attributed to *n* ≥ 4 phases because it is difficult to clarify the absorption peaks of each phase due to the shorter distance between phases with different *n* values. In contrast, the quasi-2D perovskite film on PVK, which showed the same characteristics as TFB, clearly exhibited a blue-shifted PL peak at 485 nm and excitonic absorption peaks corresponding to the *n* = 2 and *n* = 3 phases (Figure 9d). Moreover, the results of TA spectroscopy of the quasi-2D perovskite film on the TFB layer showed three distinct bleaching peaks corresponding to *n* = 2, 3, and 4 phases because of efficient energy transfer (Figure 9e). Therefore, it was confirmed that the different phase distributions depending on the underlying layers can originate from the different crystallization kinetics of the 2D and 3D phases.

### 3.3. Surface Defect Engineering of Nanoplatelets

In addition to the phase engineering strategy, the rearrangement of quasi-2D MHP phases to improve the PL and EL properties by suppressing nonradiative recombination, the synthesis of *n* = 1 2D NPL perovskites could be a promising strategy to further enhance the exciton binding energy (*E*_B_) up to 4–7 times that of 3D and quasi-2D MHPs, which have shown a small *E*_B_ of 7–16 meV, which can limit the light-emitting efficiency [73,74]. However, the exciton Bohr radius (~1.5–2.0 nm), which induces a blue-shifted emission spectrum owing to the quantum confinement effect in NPLs, is too small compared with the size of the MHP unit cell (~0.6 nm) [75,76,77]. Because of this limited crystal size requirement, a precise and reproducible NPL synthesis method is required. Furthermore, as explained above, the strong exciton–phonon coupling in 2D NPLs makes it difficult to apply them in LEDs, although they have significant advantages in terms of excitonic properties [32,53,78,79].

**Figure 10:**
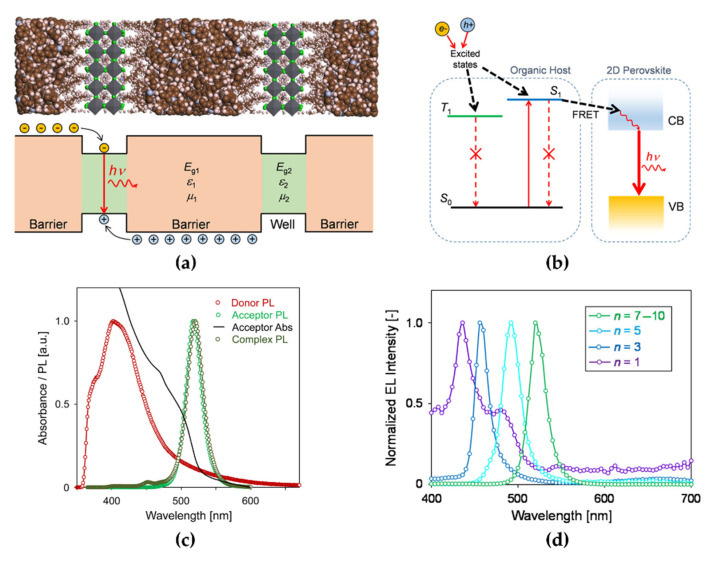
(**a**) Schematic (bottom) and molecular model (top) of the dielectric quantum wells formed between organic barriers and 2D perovskite wells. (**b**) Schematic of Förster resonance energy transfer (FRET) process occurring at the interface between the organic host and 2D perovskites (the singlet excitons in the host transferred to the conduction band [CB] of 2D perovskite). (**c**) Absorption and PL spectra of the donor (red circle), acceptor (green circle), and complex (dark green circle; the spectral overlap results in FRET from donor to acceptor). (**d**) EL spectra of 2D perovskite-based LEDs with nanoplatelet (NPL) solutions of *n* = 7−10 (pure green), *n* = 5 (sky blue), *n* = 3 (pure blue), and *n* = 1 (deep blue). Reprinted with permission from [77], Copyright 2016 American Chemical Society.

To overcome these intrinsic limitations of 2D NPL-based PeLEDs, Kumar et al. reported an organic host compound system as a dielectric quantum well matrix with dispersed 2D MAPbBr_3_ NPLs [77]. Small-bandgap 2D NPL perovskites and low-dielectric-constant (low-*k*) organic semiconductors structurally formed dielectric quantum wells as barriers (Figure 10a), which can theoretically improve *E*_B_ by up to 100 times owing to the quantum confinement effect. This *E*_B_ improvement is represented by a sharper excitonic absorption peak in the absorption spectra. Moreover, dielectric quantum well structures induce near-field Förster resonance energy transfer (FRET) at perovskite/organic barrier interfaces (Figure 10b). This is ascribed to the large spectral overlap between the absorption spectrum of the acceptor and the emission spectrum of the donor (Figure 10c). The fabricated 2D NPL PeLED showed a deep-blue EL spectrum at 432 nm with a shoulder peak at 480 nm, which can be attributed to stacking or decomposition (Figure 10d). The employed bis-4-(N-carbazolyl)phenylphosphine oxide as a low-bandgap organic host in *n* = 1 2D NPL PeLEDs reduced the self-quenching caused by adjacent 2D NPL perovskites. Consequently, they demonstrated a deep-blue 2D MAPbBr_3_ PeLED with a maximum EQE of 0.004% and a luminance of approximately 1 cd/m^2^ at room temperature.

**Figure 11:**
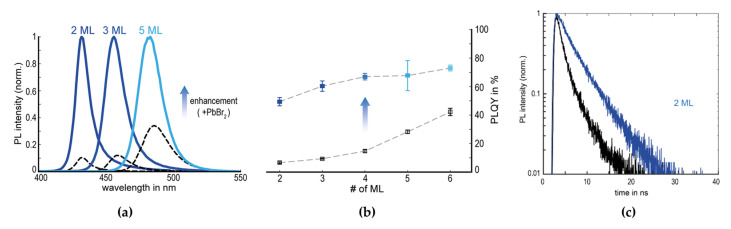
(**a**) PL spectra of initial NPL dispersions (dashed lines) and enhanced dispersions (solid lines). (**b**) PLQYs of the NPL dispersions with respect to numbers of monolayers (ML) for pre-enhancement (open black squares) and post-enhancement (full blue squares). (**c**) PL decay curves of initial (black lines) and enhanced (blue lines) NPLs. Reprinted with permission from [82], Copyright 2018 American Chemical Society.

2D NPL perovskites have shown large exciton binding energies; however, their measured PLQYs have been significantly lower than those of 3D MHPs because of the high surface-to-volume ratio of NPLs and surface traps [80,81,82]. The addition of PbBr_2_-ligands, such as oleic acid and oleylamine solution, to the NPL dispersion solution was reported as a post-treatment method to passivate the defects on the NPL surfaces [82]. The PbBr_2_-ligand treatment dramatically enhanced the PL intensities and was especially effective in the thinnest NPLs (Figure 11a). This larger enhancement in the thinnest NPLs with higher surface defects could be attributed to the higher surface-to-volume ratio, which is more susceptible to surface defects; therefore, a higher PL enhancement could be observed in two-monolayer NPL perovskites. Compared with the other bromide salt (SnBr_2_, KBr, NaBr) ligand solutions, the PbBr_2_-ligand solution was the most effective at improving the PL properties (PLQY enhancements from 7% to 49% and 42% to 73% for two and six monolayers, respectively), implying that the surfaces of NPLs have Br and Pb vacancies (Figure 11b). Moreover, the PL decay curve of the surface-treated NPLs changed from a multiexponential function to a mono-exponential function (Figure 11c). The fraction of nonradiative recombination originating from the surface defects significantly decreased, and thus, the PL decay could be dominated by radiative recombination.

**Figure 12:**
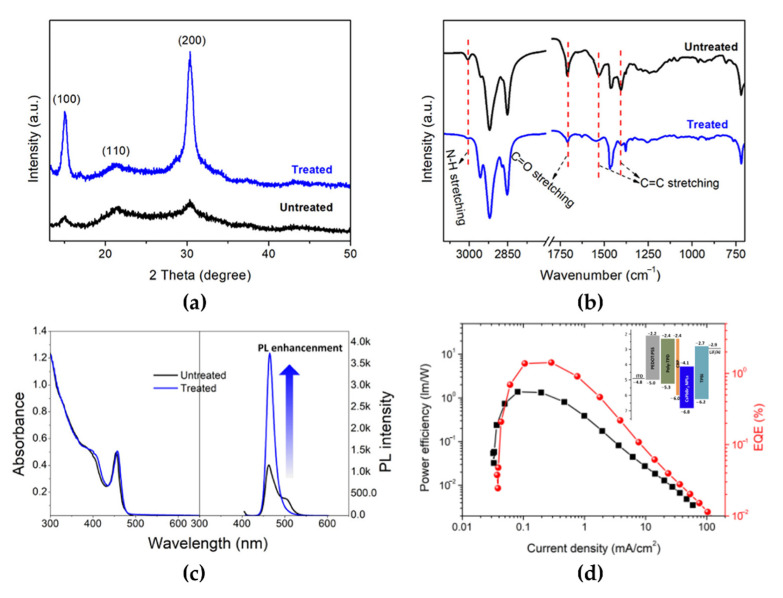
(**a**) XRD patterns, (**b**) FTIR patterns, and (**c**) absorption and PL spectra of untreated and treated CsPbBr_3_ NPLs. (**d**) Power efficiency and EQE curves as a function of current density for the treated CsPbBr_3_ NPL-based PeLEDs (inset: energy-level diagram of the CsPbBr_3_ NPLs-based PeLED with a bilayer hole transport layer structure). Reprinted with permission from [85], Copyright 2019 American Chemical Society.

Another critical issue in 2D NPL perovskites is ligand stability, which could originate from easy detachment, generating surface defects. This issue can severely deteriorate the PLQY and colloidal stability [83,84]. Congyang et al. used a halide ion-pair di-dodecyl dimethyl ammonium bromide (DDAB) ligand to improve surface ligand stability [85]. The DDAB ion-pair ligand was partially exchanged with the long-chain oleylamine ligand and passivated the surface defects without changing the crystal structure (Figure 12a). The diffraction peak intensities of the (100) and (200) planes in the DDAB-treated CsPbBr_3_ NPL clearly increased because of predominant DDA^+^ ligand passivation on these crystal planes. The sharp decrease in the absorption peaks in the FTIR spectrum at 3005, 1709, 1531, and 1404 cm^−1^ in the DDAB-treated CsPbBr_3_ NPL also indicated the exchange of oleic acid and oleylamine ligands with the DDA^+^ ligands (Figure 12b). Surface ligand passivation was clearly observed by the changes in PL properties (Figure 12c). The red shift of approximately 2 nm in the absorption and PL spectra and the significantly improved PL intensity were caused by the replacement of the DDA^+^ ligand (showing a PLQY increase from 45.1% to 69.4%). The maximum EQE and power efficiency of DDAB-treated CsPbBr_3_ NPL-based PeLEDs reached 1.42% and 1.33 lm/W, respectively (compared with 0.02% and 0.01 lm/W for the untreated CsPbBr_3_ NPL-based PeLEDs, as shown in Figure 12d). The high surface-to-volume ratio of low-*n* NPLs not only degrades the PLQY but also affects the long-term spectral stability because the FWHMs of the emission spectrum from the aggregated NPLs become broader over time [86]. To prevent NPL aggregation, effective surface-ligand control without deterioration of the conductivity of the thin film is a key issue.

**Figure 13:**
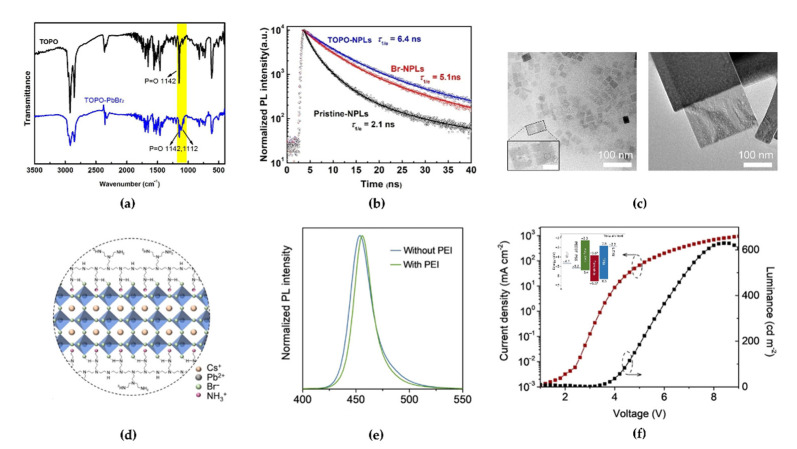
(**a**) The FTIR spectra of pure trioctylphosphine oxide (TOPO) and TOPO-PbBr_2_ adduct. Reprinted with permission from [87], Copyright 2020 American Chemical Society. (**b**) PL decay curves of pristine-NPL, Br-NPL, and TOPO−NPLs solutions. Reprinted with permission from [87], Copyright 2020 American Chemical Society. (**c**) Transmission electron microscopy images of CsPbBr_3_ NPLs without (**left**) and with (**right**) polyethylenimine (PEI), respectively (inset: a few discontinuous areas within the NPLs (**left side**) and some ~2 nm nanoparticles circled in black (**right side**)). Reprinted with permission from [89], Copyright 2021 American Chemical Society. (**d**) Schematic of CsPbBr_3_ NPLs with PEI. Reprinted with permission from [89], Copyright 2021 American Chemical Society. (**e**) PL spectra of CsPbBr_3_ NPLs with an excitation wavelength of 360 nm. Reprinted with permission from [67], Copyright 2021 American Chemical Society. (**f**) Current density (left axis) and luminance (right axis) characteristics as a function of the applied voltage in PEI–CsPbBr_3_ NPL-based PeLED (inset: energy band diagram of the PEI–CsPbBr_3_ NPL-based LED). Reprinted with permission from [89], Copyright 2021 American Chemical Society.

To overcome the low stability of low-*n* NPLs by inevitable aggregation, Peng et al. adopted trioctylphosphine oxide (TOPO) as an intermediate ligand that would not be adsorbed onto the surface of NPLs [87]. The strong coordination ability of phosphines, which act as electron-donating ligands in TOPO, could bind them to the Pb atoms and form a TOPO–PbBr_2_ adduct. A red-shifted P=O stretching vibration peak (~1112 cm^−1^) derived from the TOPO–PbBr_2_ adduct formation was observed in the FTIR spectrum (Figure 13a). The introduction of TOPO in the precursor effectively improved the reproducibility of the colloidal FAPbBr_3_ NPL solution via a ligand-tuning mechanism, and the PLQY reached 50%. Moreover, the PL peak at 444 nm remained after the washing process, and a longer PL decay lifetime of 6.4 ns (2.1 ns for pristine NPLs) was observed owing to the reduced trap density from undercoordinated metallic Pb ions and clusters, which can cause degradable reactions with negatively charged oxygen ions or moisture [88] (Figure 13b). In particular, the enhanced charge carrier mobility of TOPO-mediated NPLs was revealed through space-charge-limited current measurements of electron-only and hole-only devices. An amine-group-rich polymer can also show identical effects regarding surface defect passivation and stability of NPLs. Yin et al. used polyethylenimine (PEI) as a multidentate ligand with rich amine groups on its branched chains [89]. Transmission electron microscopy (TEM) images clearly confirmed the synthesis of stable CsPbBr_3_ NPLs using a PEI ligand (Figure 13c; left: without PEI, right: with PEI). The clear rectangular NPL crystals in the right TEM image indicate promoted crystallization and growth and low structural defects in the case of PEI introduction. In particular, the lateral size of the CsPbBr_3_ NPLs with PEI increased by more than 10 times. This effect can be explained by the fact that the branched chain of the PEI ligand can bind with Br^−^ ions, forming −NHCH_2_CH_2_NH_3_^+^·Br^−^, thereby passivating the adjacent Br vacancy defect. Moreover, hydrogen bonds of the −NH_3_^+^ moiety and secondary amine from PEI branched chains with Br^−^ ions also contributed to the stability of the NPLs. Therefore, the multidentate PEI ligand can more stably bind with NPLs compared with the monodentate oleylamine ligand because the monodentate oleylamine ligands show unrestricted movement (Figure 13d). The PL spectrum of NPLs with PEI became narrower, and the tail of the PL spectrum was reduced in the low-energy spectrum region (470–500 nm) because of the low defect density (Figure 13e). The measured lowest unoccupied molecular orbital, highest occupied molecular orbital, and Fermi energy levels of CsPbBr_3_ NPL films with PEI were –3.87, –6.57, and 4.83 eV, respectively, indicating that CsPbBr_3_ NPL with PEI could slightly show *n*-type electronic characteristics. Finally, the fabricated blue PeLEDs with PEI-derived CsPbBr_3_ NPLs exhibited stable current density–voltage–luminance characteristics with an EL spectrum at 465 nm, a peak EQE of 0.8%, and a luminance of 631 cd/m^2^ at 8.4 V (Figure 13f).

## 4. Summary

In this work, we systematically reviewed promising approaches and theoretical backgrounds for the fabrication of efficient 3D and 2D blue PeLEDs based on two main strategies: mixed-halide perovskites and low-dimensional perovskites, and we implemented multiple detailed sub-strategies for the synthesis of blue MHP emitters. Therefore, the emission mechanism of blue emitters and various engineering methods to improve the emission characteristics for each strategy were classified and analyzed.

Although many research studies have focused on overcoming the intrinsic issues of blue PeLEDs, revolutionary and creative ideas, such as the development of a novel synthesis method for blue emitters, a stable thin-film forming process, and a reliable defect passivation method should be investigated further and developed to dramatically improve device efficiency and lifetime as well as luminescent stability to match the performance level of commercial organic LEDs. In particular, long-term stability and the reproducibility of blue MHP emitters and blue PeLEDs may be the major issues that researchers need to resolve to meet industrial standards in the near future. In conclusion, this review systematically examined the core issues to be solved and potential strategies to solve them from the perspective of material engineering. We believe that this review provides comprehensive information for the development of efficient 3D and 2D blue PeLEDs and gives inspiration to the scientific community to research related perovskite issues.
